# Evolution of Serum Acute-Phase Glycoproteins Assessed by ^1^H-NMR in HIV Elite Controllers

**DOI:** 10.3389/fimmu.2021.730691

**Published:** 2021-09-28

**Authors:** Ana-Irene Malo, Joaquim Peraire, Ezequiel Ruiz-Mateos, Jenifer Masip, Núria Amigó, José Alcamí, Santiago Moreno, Josefa Girona, Graciano García-Pardo, Rosaura Reig, Francesc Vidal, Antoni Castro, Lluís Masana, Anna Rull

**Affiliations:** ^1^ Vascular Medicine and Metabolism Unit, Hospital Universitari Sant Joan, Reus, Spain; ^2^ Hospital Universitari de Tarragona Joan XXIII, Tarragona, Spain; ^3^ Infection and Immunity Research Group (INIM), Institut d’Investigació Sanitària Pere Virgili (IISPV), Reus, Spain; ^4^ Universitat Rovira i Virgili, Tarragona, Spain; ^5^ Centro de Investigación Biomédica en Red de Enfermedades Infecciosas, Instituto de Salud Carlos III (ISCIII), Madrid, Spain; ^6^ Unidad Clínica de Enfermedades Infecciosas, Microbiología y Medicina Preventiva, Instituto de Biomedicina de Sevilla-Hospital Universitario Virgen del Rocío/CSIC/Universidad de Sevilla, Sevilla, Spain; ^7^ Biosfer Teslab, Reus, Spain; ^8^ Centro de Investigación Biomédica en Red de Diabetes y Enfermedades Metabólicas Asociadas (CIBERDEM), Instituto de Salud Carlos III (ISCIII), Madrid, Spain; ^9^ AIDS Immunopathogenesis Unit, Instituto de Salud Carlos III, Madrid, Spain; ^10^ HIV Unit, Hospital Clinic-Institut d'Investigacions Biomèdiques August Pi iSunyer (IDIBAPS), Barcelona, Spain; ^11^ Hospital Universitario Ramón y Cajal Universidad de Alcalá, Instituto Ramón y Cajal de Investigación Sanitaria (IRYCIS), Madrid, Spain; ^12^ Research Unit on Lipids and Atherosclerosis, Institut d’Investigació Sanitària Pere Virgili (IISPV), Reus, Spain

**Keywords:** elite controllers, HIV, inflammation, acute-phase glycoproteins, proton nuclear magnetic resonance

## Abstract

Elite controllers (ECs) are an exceptional group of people living with HIV (PLWH) who maintain undetectable viral loads (VLs) despite not being on antiretroviral therapy (ART). However, this phenotype is heterogeneous, with some of these subjects losing virological control over time. In this longitudinal retrospective study, serum acute-phase glycoprotein profile assessed by proton nuclear magnetic resonance (^1^H-NMR) was determined in 11 transient controllers (TCs) who spontaneously lost virological control and 11 persistent controllers (PCs) who persistently maintained virological control over time. Both PCs and TCs showed similar acute-phase glycoprotein profiles, even when TCs lost the virological control (GlycB, p = 0.824 and GlycA, p = 0.710), and the serum acute-phase glycoprotein signature in PCs did not differ from that in HIV-negative subjects (GlycB, p = 0.151 and GlycA, p = 0.243). Differences in serum glycoproteins A and B were significant only in ECs compared to HIV-typical progressors (TPs) with < 100 CD4+ T-cells (p < 0.001). ^1^H-NMR acute-phase glycoprotein profile does not distinguish TCs form PCs before the loss of viral control. ECs maintain a low-grade inflammatory state compared to TPs. PCs revealed a closer serum signature to HIV-negative subjects, reaffirming this phenotype as a closer model of functional control of HIV.

## Introduction

Elite controllers (ECs) are a select group of people living with HIV (PLWH) who maintain a circulating viral load (VL) at undetectable levels without antiretroviral treatment (ART) (1, 2). This exceptional characteristic makes ECs a good pathogenic model for the functional control of HIV (3, 4). However, ECs consist of a heterogeneous population in terms of virological, immunological and clinical outcomes over time (5–7). Some of them maintain strong virological and immunological control for years and are called persistent controllers (PCs) while others lose virological control over time and are known as transient controllers (TCs).

Although it was once thought that ECs could have a favorable short- and long-term prognosis, recent real-life data have revealed that ECs are at increased risk of several non-AIDS events (7). Notably it is that most of these events have been pathogenically related to the subclinical low-level inflammatory state due to HIV itself (8). This fact provides the rationale behind the recommendation for initiating ART in ECs suggested by some authorities to reverse this subclinical inflammatory state (5). With respect to this issue, a major weakness is the lack of appropriate inflammatory biomarkers that could predict low-level inflammation in the long term. The inflammatory state in PLWH is usually assessed through the determination of C-reactive protein (PCR) or IL-6 in plasma, among other biomarkers (9). These molecules are easy to assess but are labile and subject to large variations if new conditions occur; hence, although useful, several limitations do exist that decrease their value.

In recent years serum glycoproteins have emerged as potential markers of inflammation-associated diseases (10–13). The composite nature of ^1^H-NMR serum glycoprotein measurement encompasses the systemic inflammatory process more comprehensively than other individual biomarkers (14, 15). It is a technique that globally identifies glycosylated proteins, which increase their concentration and modify their structure during inflammatory processes. Thus, a small subset of various acute phase glycoproteins that are more abundant in circulation (16, 17) makes meaningful contributions to the glycoprotein A (GlycA) signal and glycoprotein B (GlycB) signal. Of note, previous studies suggested that GlycA better captures systemic inflammation even more than C-reactive protein (CRP), a widely used classical inflammatory marker (18). Circulating GlycA elevations in inflammatory states could reflect the increased concentrations of 1-acid glycoprotein, haptoglobin, 1-antitrypsin, 1-antichymotrypsin, and transferrin. Hence, the advantage offered by Glyc A than the conventional CRP is that it may integrate more multiple inflammatory pathways by capturing the global signal of several proteins and, therefore, better captures the degree of systemic inflammation. On the other hand, the measurement of GlycA presents higher reliability and lower intra-individual variability because its measures are similar in both serum and plasma samples, in fasting and non-fasting states, and also after short or long-term storage (18).

We and others have previously shown serum glycoproteins to be useful diagnostic biomarkers in PLWH (19–21). The baseline serum glycoprotein signature by ^1^H-NMR predicted the immunological response to ART in typical HIV progressors, emphasizing the role of inflammation mediators in poor recovery status (19). Here, we analyze the evolution of serum acute-phase glycoproteins in a cohort of ECs containing both PCs and TCs, compared to a group of HIV-typical progressors and a group of healthy HIV-negative participants. We aimed to evaluate whether there is altered expression of these inflammation-related molecules associated with phenotypic evolution in ECs. Understanding the molecular pathways associated with ECs heterogeneity would be crucial to balance the risks and benefits of ART in ECs.

## Materials and Methods

### Study Design and Participants

According to the study design, frozen serum samples from the Spanish HIV HGM biobank belonging to the AIDS Research Network (RIS) (22) were included and the data were recorded in the RIS cohort of the HIV Controllers Study Group (ECRIS) (5).

Twenty-two elite controllers (ECs) were selected, defined as subjects who in the absence of prior or current ART since HIV diagnosis and during the 36 months of the follow-up maintained an undetectable VL. Two groups were distinguished: 11 ECs who lost spontaneous viral load control, defined by detection at least two consecutive measurements above 50 HIV-RNA copies/mL within a year (TCs); and 11 ECs who maintained virological control throughout the follow-up (PCs), as previously described (23).

**Figure 1:**
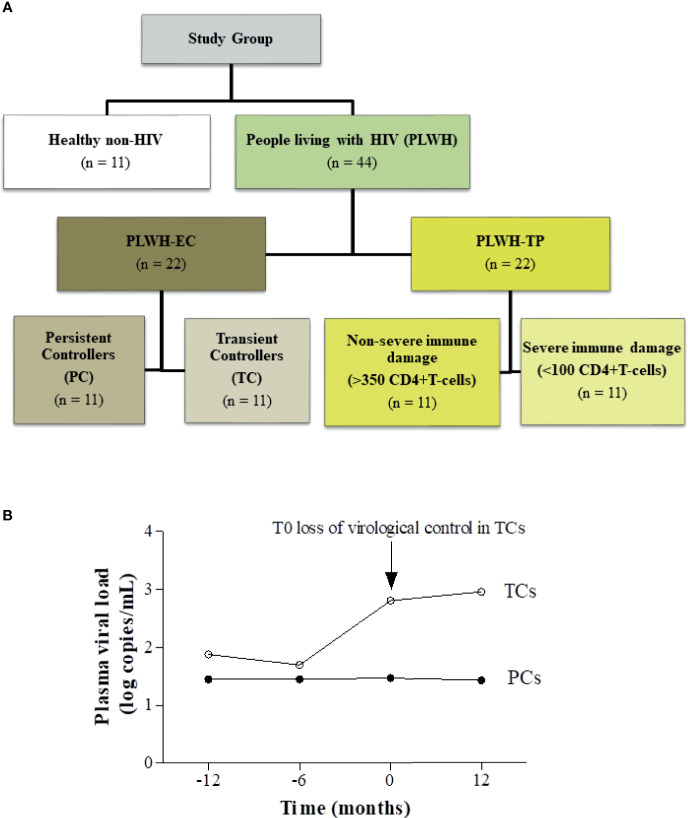
Study design. **(A)** Flow chart illustrating subject cohort enrolment and analysis. People living with HIV (PLWH), grouped into elite controllers (PLWH-EC) and typical progressors (PLWH-TP), were compared to a group of healthy non-HIV subjects. The PLWH-EC group was grouped according to their virological progression as persistent controllers (PCs) if they maintained virological control over time or as transient controllers (TCs) when they lost the spontaneous viral load during at least two consecutive measurements during one year. The PLWH-TP group was also categorized according to their pre-cART CD4^+^ T-cell counts in subjects presenting nonsevere immune damage (>350 CD4^+^ T-cells) or subjects with severe immune damage (<100 CD4^+^ T-cells). **(B)** Schematic representation indicating the follow-up time point of the study design in PLWH-EC. The arrow indicates T0, the time point closest the time when TCs lost the virological control, which was considered the baseline.

Regarding the glycoprotein profile, repeated analyses were performed during the follow-up in TCs: two years before the loss of virological control, one year before the loss of virological control (T-1), 6 months before the virological control, at the moment when viral load was detected (T0) and subsequently at one year after the spontaneous loss of control (T+1). In PCs, repeated analyses were also performed at least at two different time points, but no differences were found in the glycoprotein profiles during any of the follow-ups. Thus, median values were calculated and identified as T0 for the PC group (**Figure 1**).

Two other cohorts of patients were included in the study: healthy HIV-negative-subjects and PLWH with a typical disease progression pattern (TP), matched by sex and age with ECs (24). For the PLWH-TP group, eleven patients were defined as HIV-typical progressors with severe immunosuppression (< 100 CD4^+^ T-cells at starting ART) and HIV-typical progressors with non-severe immunosuppression (> 350 CD4+ T-cells at starting ART) (**Figure 1**) (24). Serum glycoproteins for PLWH-TP were analyzed at baseline before ART onset (T0).

All the selected patients had be age over 18 years and no had concomitant acute or chronic conditions or was taking drugs (NSAID, steroids, immunomodulators) that could eventually modify the inflammatory status when the blood samples were collected.

### Glycoprotein Analysis by ^1^H-NMR

Samples were prepared and analysed by ^1^H-NMR for glycoprotein profiling as previously reported (12, 19). The resonance spectra were recorded at 310 K in a Bruker Avance III 600 spectrometer at a proton frequency of 600.20 MHz (14.1 T). GlycA and GlycB are different functions depending on the chemical changes in the glycoproteins that resonate in a region of the spectrum analysed at 2.15-1.90 ppm. The total area of each function, which is translated into a concentration according to the number of sugar-protein bonds, was analysed. The number of acetyl groups of the bonds of N-acetylglucosamine and N-acetylgalactosamine, and N-acetylneuraminic acid is reflected by the concentration of Glyc A and GlycB, respectively. The associated height/width ratios of GlycA and GlycB (H/W) were also calculated. They are associated with the peaks of the signals that are generated by 1H-NMR and that reflect the state of aggregation or the flexibility of the sugar-protein bonds (12).

### Statistical Analysis

The continuous variables presented a non-normal distribution due to the small sample size. Data are expressed as medians and 25th and 75th percentiles. Categorical variables are expressed as percentages. Differences between two groups were analysed using the nonparametric Mann-Whitney test, and for multiple comparisons one- way ANOVA followed by Bonferroni’s *post hoc* test was applied. A p value less than 0.05 was considered statistically significant. Statistical analyses were performed with SPSS software, version 25 (IBM, Madrid, Spain), and graphical representations were generated with GraphPad Prism software (version 5.0, GraphPad Inc., San Diego, CA, USA).

### Ethics

The study and all of the research protocols were conducted in accordance with the recommendations of the Ethical and Scientific Committees, and all of them were approved by the Committee for Ethical Clinical Research by following the rules of Good Clinical Practice from the Institut d’Investigació Sanitària Pere Virgili (CEIM 041/2019). The CEIm IISPV is an independent committee, consisting of health and non-health professionals that supervise the correct compliance to the ethical principles governing clinical trials and research projects that are performed in our region, specifically in terms of methodology, ethics and laws. An informed consent form signed by each participant was obtained in accordance with the Declaration of Helsinki and which includes the allowance to use the samples stored in the Biobank for related biomedical research projects.

## Results

### Patient Characteristics

A flow chart illustrating patient enrolment and categorization is shown in **Figure 1A**. **Figure 1B** details a schematic representation of the study design and categorization in PLWH-EC. Clinical characteristics at T0 are presented in **Table 1**. At that time, defined by the loss of control in TC subjects, no differences were observed in age, sex, transmission route, HCV coinfection, CD4^+^ T-cell or CD8^+^T-cell counts between TCs and PCs. The T0 since HIV diagnosis was not statistically significance between TCs than PCs (P=0.061).

**Table 1:** Clinical characteristics of the study cohort.

	PLWH-EC		P-value*	PLWH-TP		Non-HIV (n = 11)	P-value**
	PCs (n = 11)	TCs (n = 11)		>350 (n = 11)	<100 (n = 11)		
Age (years)	47 [43-50]	45 [38-56]	0.605	43 [37-47]	42 [34-48]	52 [36-53]	0.502
Male, n (%)	6 (55)	6 (55)	0.612	8 (73)	7 (63)	7 (63)	0.898
Risk factor, n (%)			0.370			–	0.012
Heterosexual	3 (27)	4 (36)		5 (45)	8 (73)		
Homo/Bisexual	2 (18)	2 (18)		6 (55)	2 (18)		
Intravenous drug abuse	6 (55)	3 (27)		–	1 (9)		
Other/Unknown	–	2 (18)		–	–		
Time since diagnosis (years)	18 [13-23]	10 [5-19]	0.061	–	–	–	–
HCV RNA detected, n (%)	4 (36)	5 (45)	0.856	–	2 (18)	–	0.001
CD4+ T-cell count (cells/µL)	651 [442-950]	720 [414-951]	0.748	408 [371-578]	38 [7-62]	–	<0.001
CD8+ T-cell count (cells/µL)	768 [553-1082]	743 [945-1224]	0.387	1217 [900-1570]	669 [400-925]	–	<0.001
CD4:CD8 ratio	1.1 [0.5-1.4]	0.8 [0.6-1.2]	0.557	0.5 [0.3-0.6]	0.1 [0.1-0.2]	–	<0.001

T0 in PLWH-ECs is defined by the loss of control in transient controllers (TCs) and the initiation of ART in PLWH-TP. Data are presented as n (%) or median (interquartile range). *Categorical data were compared by means of a χ2 test, whereas continuous data were compared using non-parametric Mann-Whitney test between PCs and TCs*, and between all PLWH-EC, PLWH-TP and non-HIV subjects with nonparametric Kruskal-Wallis (KW) adjusted by Bonferroni post-hoc approach **. P value < 0.05 was considered significant. EC, Elite controller; Glyc, glycoprotein; H/W, height/width glycoprotein ratio; PC, Persistent controller; PLWH, Person living with HIV; TC, Transient controller.

### Stability in the Serum Glycoprotein Profile Before the Loss of Viral Control

To predict the loss of control in TCs, serum glycoprotein concentration and H/W ratios analyzed at 24 months, 12 months and 6 months before the loss of control in TCs were compared to T0 values in the PC group. No differences were observed among the groups of studies and thus, we selected data from 12 months before the loss of control (T-1) as the representative value for TCs (**Table 2**). Then, the serum glycoprotein profile at time when viral load was detected in TCs (T0) was also compared to T0 from the PC group. Acute-phase glycoprotein analysis revealed similar serum glycoprotein A (Glyc A) and B (Glyc B) concentrations, as well as H/W ratios, in TCs and PCs (**Table 2**). However, at the point when TCs revealed viral rebound, the H/W GlycA ratio showed a significant, positive association with VL in this group of patients (ρ=0.683, P= 0.042).

**Table 2:** Plasma glycoprotein analysis in PLWH-EC.

	PC	TC		P-value*	
	T0 (n = 11)	T-1 (n = 10)	T0 (n = 11)	PC T0 *vs* TC T-1	PC T0 *vs* TC T0
Glyc B (µmol/L)	400.8 [356.0-443.5]	410.9 [375.1-455.5]	404.0 [373.6-462.5]	0.756	0.824
Glyc A (µmol/L)	746.2 [638.7-887.8]	795.3 [706.2-848.5]	789.1 [718.9-914.2]	0.918	0.710
H/W Glyc A	5.0 [4.5-5.6]	5.2 [4.7-5.7]	5.1 [4.7-5.8]	0.756	0.824
H/W Glyc B	18.3 [15.9-20.8]	18.7 [17.7-20.8]	20.1 [17.8-20.8]	0.426	0.331

T0 in PC shows median values from at least two different time-points during the follow-up in this group of study. In TC, T-1 corresponds to one year before the loss of viral control and T0 is defined by the closest moment where viral load was detected. *Data was compared using non-parametric Mann-Whitney test between PC and TC in each different time-point for TC.

Glyc, glycoprotein; H/W, height/width glycoprotein ratio; PC, Persistent controller; PLWH-EC, Person living with HIV defined as elite controller; TC, Transient controller.

### Serum Glycoprotein Signature in PCs Closer to Healthy HIV-Negative Individuals

**Figure 2:**
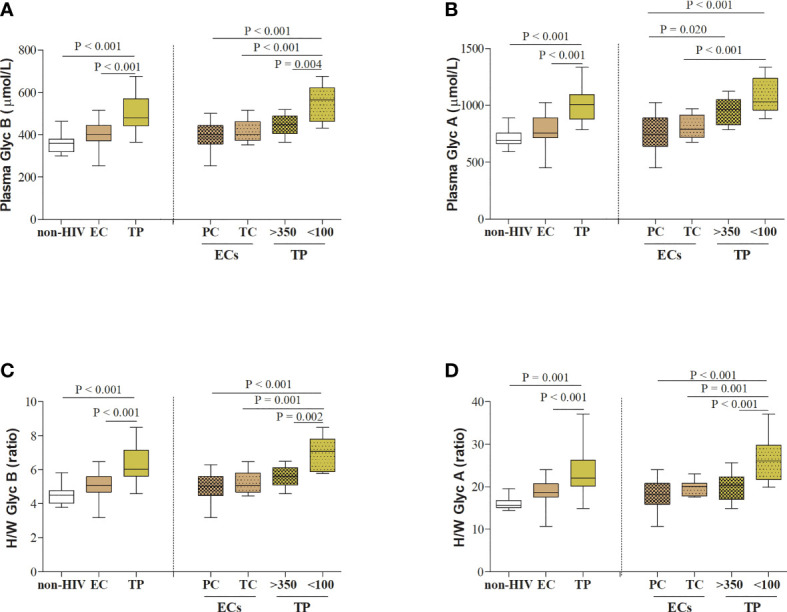
Serum acute-phase glycoprotein profile in PLWH. **(A)** Serum glycoprotein B and **(B)** glycoprotein A concentrations, and **(C)** height/width ratios for glycoprotein B and **(D)** glycoprotein A are represented for HIV-elite controllers (ECs) and HIV-typical progressors (TPs) and healthy HIV-negative individuals (n=11), matched by sex and age. PLWH in each different cohort of HIV studies (PLWH-EC and PLWH-TP) were also classified according to the viral control or the pre-cART CD4+ T-cell counts, respectively. For PLWH-EC, data for PCs corresponded to median values from at least two different time points (T0), and data for TCs corresponded to the closest moment to the loss of viral control. For PLWH-TP, data from T0 corresponded to the moment when subjects were enrolled in the study and started their first ART (pre-ART). Thus, PLWH-TP were classified in subjects presenting nonsevere immune damage (>350 CD4+ T-cells) or subjects with severe immune damage (<100 CD4+ T-cells). Statistical analysis was performed by one-way ANOVA followed by Bonferroni’s *post hoc* test. Data are re-presented as boxes and whiskers (min to max values). EC, elite controller; Glyc, glycoprotein; H/W, height/width glycoprotein ratio; non-HIV, healthy HIV-negative individuals; PC, persistent controller; PLWH, people living with HIV; TC, transient controller; TP, typical progressor.

Next, serum acute-phase glycoproteins in PCs and TCs at T0 were compared to a control group of healthy HIV-negative individuals (n=11), matched by sex and age (**Figure 2** and **Supplemental Table 1**). Serum Glyc B and Glyc A concentrations and H/W Glyc B and Glyc A ratios were lower in HIV-negative subjects than in the PLWH-EC group (GlycB, 358.8 *vs.* 402.4, respectively, P = 0.044; GlycA, 691.4 *vs.* 757.5, respectively, P= 0.054; H/W Glyc B, 4.5 *vs.* 5.1, respectively, P = 0.049; H/W Glyc A, 15.7 *vs.* 18.7, respectively, P = 0.002). However, when the serum glycoprotein signature in healthy HIV-negative subjects was separately compared to each group of PLWH-EC, only the difference in TCs remained significant compared to the group of healthy HIV-negative subjects (**Figure 2** and **Supplemental Table 1**). PC did not show a glycoprotein profile significantly different from HIV-negative subjects.

### Similar Glycoproteins for PLWH-EC and Typical Progressors With > 350 CD4^+^ T-Cells

We included in the present work 22 PLWH-TPs coming from a previously described cohort (24). For this study, a group of typical HIV-progressors (PLWH-TP) were matched by sex (68% male, P = 0.268) and age (42 [37-47], P = 0.78) with the study cohort of PLWH-EC at T0. As expected, PLWH-TP showed lower CD4^+^ T-cell counts and increased VL compared to PLWH-EC (CD4^+^ T-cell counts 272.0 [31.5-443.0] cells/µL in TPs, P= 0.001 and 5.46 [4.61-5.60] log copies/mL in TP, P<0.001). PLWH-TP showed higher serum glycoprotein concentrations and H/W ratios than the PLWH-EC group (**Figure 2**): GlycB, 478.9 *vs.* 402.4, respectively, P <0.001; GlycA, 1007.9 *vs.* 757.5, respectively, P < 0.001; H/W Glyc B, 6.02 *vs.* 5.06, respectively, P < 0.001; H/W Glyc A, 22.05 *vs.* 18.68, respectively, P = 0.001.

When PLWH-TP were grouped according to their CD4^+^ T-cell counts at T0 (starting cART), serum Glyc A and Glyc B concentration and H/W Glyc A and Glyc B ratios resulted significantly higher in TP with < 100 CD4^+^ T-cells compared to both, PC and TCs (**Figure 2**). This was not the case for the group of TP with > 350 CD4^+^ T-cells, in which no differences were found in serum glycoprotein profile except for GlycA concentration when compared with PC P = 0.020 (**Figure 2**).

### Longitudinal Glycoprotein Profile in TC, From Viral Control to Viral Rebound

**Figure 3:**
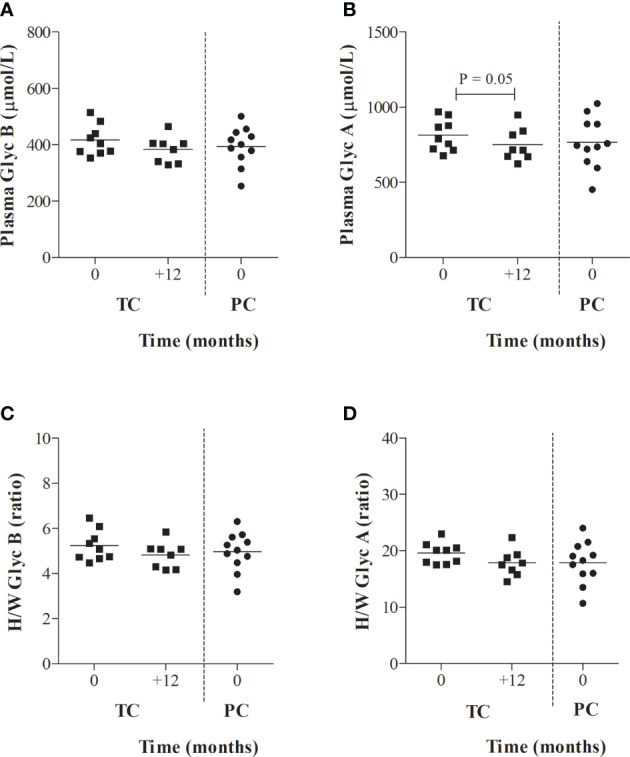
Serum glycoprotein evolution in TCs compared to PCs. **(A)** Serum glycoprotein B and **(B)** glycoprotein A concentrations and height/width ratios for **(C)** glycoprotein A and **(D)** glycoprotein B are represented for transient controllers (TCs) during their follow-up. Values from PCs obtained in **Figure 2** at T0 were also included as reference values for TCs. Data were compared using the Wilcoxon’s non-parametric test for paired samples.

Serum glycoprotein evolution in TC, from 24 months before to one year after the loss of virological control (T+1), was also evaluated in a longitudinal analysis. VL evolution from TC after one year of loss of control was not significantly different from baseline (T0) (3.24 [2.30-4.00] log copies/mL at T+1, P =0.612) or the decrease in the CD4+ T-cell counts (516.0 [407.5-835.0] log copies/mL at T+1, P =0.499). However, TC showed a slightly decreased acute-phase glycoprotein profile at one year compared to T0, although the results were only significant for serum GlycA concentrations (P = 0.05). In fact, after one year of viral rebound (T+1), the serum glycoprotein profile in TCs (716.7 [671.4-835.5] µmol/L) became more similar to the serum glycoprotein profile of PCs at T0 (746.2 [638.7-887.8] µmol/L), although maintained a sustained low-grade inflammation in PLWH-EC compared to HIV-negative individuals (691.4 [664.8 – 755.5] µmol/L) (**Figure 3**).

## Discussion

The loss of spontaneous viral control in TC has been extensively investigated. According to different publications, it seems that there is no single mechanism responsible for controlling viral replication. This situation could be associated with genetic factors in the host (mainly HLA class I, such as HLA-B * 57) (25), low polyfunctionality of Gag-specific T cells (26) alteration of T cell homeostasis (6), high viral diversity (26), the suppressive capacity of HIV-1 (27) and immunological mechanisms (elevated inflammatory cytokines (28), and chemokines and cytolytic enzymes as a response of the specific T cells of HIV-1). In this sense, a specific proteomic signature that analysed proteins involved in pro-inflammatory pathways (clotting factor XI, alpha-1-antymotrypsin, ficolin-2, protein 14-3-3, and galectin-binding protein-3 were considered potential biomarkers) predicted that PLWH-EC would lose control over viral load (29).

In this work, we studied the serum glycoprotein profile determined by ^1^H-NMR in PLWH-EC. Low-grade inflammation seems to remain present in PLWH-EC and is one of the reasons why the use of antiretrovirals is debated despite spontaneous suppression of the virus. In our previous work, glycoproteins were prognostic of immune recovery in PLWH (19) and were shown to be a true reflection of the inflammatory state. This finding is based on an increase in the concentration and structure of glycosylated proteins in inflammatory states, increasing the branching and number of monosaccharide residues, including N-acetylglucosamine, N-acetylgalactosamine (GlycA) and N-acetylneuraminic acid (GlycB) among others (29). They are considered more stable inflammatory markers since they are compounds of several different acute phase proteins (transferrin, haptoglobin, α1 acid glycoprotein, α1 antitrypsin, α1 antitrypsin) (18). In fact, the measurement of glycoproteins presents high reliability and low intra-individual variability because its measures are similar in both serum and plasma samples, in fasting and non-fasting states, and also after short or long-term storage (30).

In the present study, we observed that the glycoprotein profile remained the same between PC and TC individuals prior to the spontaneous loss of viral control. However, a significant association of H/W GlycA was observed with viral load in TCs once they lost viral control (T0). This finding is in line with previous publications (19) in which glyco-proteins showed a marked association with viral load. PLWH-EC presented glycoprotein levels higher than those of healthy non-HIV individuals. However, when compared separately, PC and healthy non-HIV individuals did not show differences in the glycoprotein profiles, demonstrating an inflammatory status of PCs similar to that of uninfected individuals. Only TCs showed differences in glycoprotein levels compared to healthy patients. This finding reflects the low-grade inflammation that occurs in parallel with the loss of spontaneous viral control and is responsible for non-AIDS comorbidities (8). The presence of low-grade inflammation in the absence of suppression of viral replication is also evidenced when comparing PLWH-TP with PLWH-EC, in which the concentration of glycoproteins and their proportions were higher in the PLWH-TP (the latter had higher CV and lower CD4^+^ levels). In short, as seen in previous publications, a higher level of glycoproteins will be associated with decreased CD4 counts and higher viral load. This outcome is also reflected when separating the TPs into subgroups, in which the subgroup with the best immune status (CD4+ T-cell> 350) presented a glycoprotein profile more similar to the PLWH-EC compared to the group of PLWH-TP with severe immune damage (CD4^+^ T-cell <100 cells/µL).

The main limitation of the study is the small number of subjects in each arm of the study design which probably means there was insufficient power to detect significant predictive value of the GlycA and B measures, since in previous studies they did predict immune recovery (19). However, elite controllers are an exceptional population within PLWH, and small sample sizes are common in these population studies. The relation to other HIV cohorts and HIV negatives (healthy volunteers) was used to enhance the information regarding the ECs. And since the statistical power is highest when the groups have equal sample sizes, we selected the same number of participant for the other subsets. But larger cohort studies are needed to establish whether 1H-NMR glycoproteins might be of value in predicting loss of spontaneous viral control. Another limitation was lack of data presented on potential confounders for measures of inflammation, particularly tobacco use (31).

## Conclusions

High concentrations of glycoproteins determined by ^1^H-NMR have been associated, within the same disease, with worse states (29). To the best of our knowledge, this study is the first with elite controllers that provides an insight into their inflammatory status using this novel method of ^1^H-NMR glycoprotein determination. In this work, we observed that glycoproteins remain at normal levels or are elevated depending on the viral control and immunological status of the patients.

## Data Availability Statement

The raw data supporting the conclusions of this article will be made available by the authors, without undue reservation.

## Ethics Statement

The studies involving human participants were reviewed and approved by Comité Ético de Investigación con Medicamentos del Insitut d’Investigació Sanitària Pere Virgili (CEIm-IISPV). The patients/participants provided their written informed consent to participate in this study.

## Author Contributions

All authors contributed to the article and approved the submitted version. The authors contributions are as follows: experimental design (AIM, JM, FV, ER-M, LM, and AR) and intellectual guidance (JA, SM, JG, RR, and AC). recruitment of subjects (JP and ER-M) and sample procurement (AIM and JM). Data collection (AIM and NA). data analysis and interpretation (AIM, JM, and AR). manuscript preparation (AIM and JM). AI-M, JM, AC, FV, LM, and AR were responsible for the study design, data analysis, and article development. FV, LM and AR reviewed and edited the manuscript.

## Funding

This research was funded by the Fondo de Investigacion Sanitaria [PI16/00503, PI19/01337 and PI20/00326]-ISCIII-FEDER (co-funded by the European Regional Development Fund/European Social Fund; “A way to make Europe”/“Investing in your future”); Programa de Suport als Grups de Recerca AGAUR (2017SGR948); the SPANISH AIDS Research Network [RD12/0017/0005, RD12/0025/0001, RD16/0025/0006]-ISCIII-FEDER (Spain) and Centro de Investigación Biomédica en Red de Enfermedades Infecciosas-ISCIII [CB21/13/00015, CB21/13/00020, CB13/21/00086], Madrid, Spain. JM is supported by the Universitat Rovira i Virgili under grant agreement “2019PMF-PIPF-18,” through the call “Martí Franquès Research Fellowship Programme”. FV is supported by grants from the Programa de Intensificación de Investigadores (INT20/00031)-ISCIII. AR is supported by a grant from IISPV through the project “2019/IISPV/05” (Boosting Young Talent), by GeSIDA through the “III Premio para Jóvenes Investigadores 2019” and by the Instituto de Salud Carlos III (ISCIII) under grant agreement “CP19/00146” through the Miguel Servet Program.

## Conflict of Interest

NA is stockowner of Biosfer Teslab, the company that commercializes the glycoprotein profiling described in the present manuscript.

The remaining authors declare that the research was conducted in the absence of any commercial or financial relationships that could be construed as a potential conflict of interest.

## Publisher’s Note

All claims expressed in this article are solely those of the authors and do not necessarily represent those of their affiliated organizations, or those of the publisher, the editors and the reviewers. Any product that may be evaluated in this article, or claim that may be made by its manufacturer, is not guaranteed or endorsed by the publisher.

## References

[1] Deeks SG Walker BD. Human Immunodeficiency Virus Controllers: Mechanisms of Durable Virus Control in the Absence of Antiretroviral Therapy. Immunity (2007) 27:406–16. doi: 10.1016/j.immuni.2007.08.010 17892849

[2] Navarrete-Muñoz MA Restrepo C Benito JM Rallón N. Elite Controllers: A Heterogeneous Group of HIV-Infected Patients. Virulence (2020) 11:889–97. doi: 10.1080/21505594.2020.1788887 PMC7549999 32698654

[3] Casado C Galvez C Pernas M Tarancon-Diez L Rodriguez C Sanchez-Merino V. Permanent Control of HIV-1 Pathogenesis in Exceptional Elite Controllers: A Model of Spontaneous Cure. Sci Rep (2020) 10:1902. doi: 10.1038/s41598-020-58696-y 32024974 PMC7002478

[4] Lopez-Galindez C Pernas M Casado C Olivares I Lorenzo-Redondo R. Elite Controllers and Lessons Learned for HIV-1 Cure. Curr Opin Virol (2019) 38:31–6. doi: 10.1016/j.coviro.2019.05.010 31252326

[5] Dominguez-Molina B Leon A Rodriguez C Benito JM Lopez-Galindez C Garcia F. Analysis of Non-AIDS-Defining Events in HIV Controllers. Clin Infect Dis (2016) 62:1304–9. doi: 10.1093/cid/ciw120 26936669

[6] Benito JM Ortiz MC León A Sarabia LA Ligos JM Montoya M. Class-Modeling Analysis Reveals T-Cell Homeostasis Disturbances Involved in Loss of Immune Control in Elite Controllers. BMC Med (2018) 16:30. doi: 10.1186/s12916-018-1026-6 29490663 PMC5830067

[7] Ruiz-Mateos E Poveda E Lederman MM. Antiretroviral Treatment for HIV Elite Controllers? Pathog Immun (2020) 5:121–33. doi: 10.20411/pai.v5i1.364 PMC7307444 32582872

[8] Sokoya T Steel HC Nieuwoudt M Rossouw TM. HIV as a Cause of Immune Activation and Immunosenescence. Mediators Inflammation (2017) 2017:6825493. doi: 10.1155/2017/6825493 PMC5676471 29209103

[9] Justice AC Erlandson KM Hunt PW Landay A Miotti P Tracy RP. Can Biomarkers Advance HIV Research and Care in the Antiretroviral Therapy Era? J Infect Dis (2018) 217:521–8. doi: 10.1093/infdis/jix586 PMC5853399 29165684

[10] Gruys E Toussaint MJM Niewold TA Koopmans SJ. Acute Phase Reaction and Acute Phase Proteins. J Zhejiang University Sci B (2005) 6:1045–56. doi: 10.1631/jzus.2005.B1045 PMC1390650 16252337

[11] Gornik O Lauc G. Glycosylation of Serum Proteins in Inflammatory Diseases. Dis Markers (2008) 25:267–78. doi: 10.1155/2008/493289 PMC3827815 19126970

[12] Fuertes-Martín R Taverner D Vallvé J-C Paredes S Masana L Correig Blanchar X. Characterization of 1 H NMR Plasma Glycoproteins as a New Strategy To Identify Inflammatory Patterns in Rheumatoid Arthritis. J Proteome Res (2018) 17:3730–39. doi: 10.1021/acs.jproteome.8b00411 30353728

[13] Fuertes-Martín R Moncayo S Insenser M Martínez-García MÁ Luque-Ramírez M Grau NA. Glycoprotein A and B Height-To-Width Ratios as Obesity-Independent Novel Biomarkers of Low-Grade Chronic Inflammation in Women With Polycystic Ovary Syndrome (PCOS). J Proteome Res (2019) 18:4038–45. doi: 10.1021/acs.jproteome.9b00528 31503497

[14] Duprez DA Otvos J Sanchez OA Mackey RH Tracy R Jacobs DR. Comparison of the Predictive Value of GlycA and Other Biomarkers of Inflammation for Total Death, Incident Cardiovascular Events, Noncardiovascular and Noncancer Inflammatory-Related Events, and Total Cancer Events. Clin Chem (2016) 62:1020–31. doi: 10.1373/clinchem.2016.255828 27173011

[15] Akinkuolie AO Buring JE Ridker PM Mora SA. Novel Protein Glycan Biomarker and Future Cardiovascular Disease Events. J Am Heart Assoc (2014) 3:e001221. doi: 10.1161/JAHA.114.001221 25249300 PMC4323825

[16] Gabay C Kushner I. Acute-Phase Proteins and Other Systemic Responses to Inflammation. N Engl J Med (1999) 340:448–54. doi: 10.1056/NEJM199902113400607 9971870

[17] Ohtsubo K Marth JD. Glycosylation in Cellular Mechanisms of Health and Disease. Cell (2006) 126:855–67. doi: 10.1016/j.cell.2006.08.019 16959566

[18] Malo A-I Rull A Girona J Domingo P Fuertes-Martín R Amigó N. Glycoprotein Profile Assessed by 1H-NMR as a Global Inflammation Marker in Patients With HIV Infection. A Prospective Study. J Clin Med (2020) 9:1344. doi: 10.3390/jcm9051344 PMC7291035 32375373

[19] Otvos JD Shalaurova I Wolak-Dinsmore J Connelly MA Mackey RH Stein JH. GlycA: A Composite Nuclear Magnetic Resonance Biomarker of Systemic Inflammation. Clin Chem (2015) 61:714–23. doi: 10.1373/clinchem.2014.232918 25779987

[20] Kelesidis T Tran TTT Stein JH Brown TT Moser C Ribaudo HJ. Changes in Inflammation and Immune Activation With Atazanavir-, Raltegravir-, Darunavir-Based Initial Antiviral Therapy: ACTG 5260s. Clin Infect Dis (2015) 61:651–60. doi: 10.1093/cid/civ327 PMC4542595 25904376

[21] Tibuakuu M Fashanu OE Zhao D Otvos JD Brown TT Haberlen SA. GlycA, a Novel Inflammatory Marker, Is Associated With Subclinical Coronary Disease. AIDS (2019) 33:547–57. doi: 10.1097/QAD.0000000000002079 PMC6559349 30475263

[22] García-Merino I de Las Cuevas N Jiménez JL Gallego J Gómez C Prieto C. Spanish HIV BioBank The Spanish HIV BioBank: A Model of Cooperative HIV Research. Retrovirology (2009) 6:27. doi: 10.1186/1742-4690-6-27 19272145 PMC2667474

[23] Tarancon-Diez L Rodríguez-Gallego E Rull A Peraire J Viladés C Portilla I. Immunometabolism Is a Key Factor for the Persistent Spontaneous Elite Control of HIV-1 Infection. EBioMedicine (2019) 42:86–96. doi: 10.1016/j.ebiom.2019.03.004 30879922 PMC6491381

[24] Yeregui E Viladés C Domingo P Ceausu A Pacheco YM Veloso S. High Circulating SDF-1and MCP-1 Levels and Genetic Variations in CXCL12, CCL2 and CCR5: Prognostic Signature of Immune Recovery Status in Treated HIV-Positive Patients. EBioMedicine (2020) 62:103077. doi: 10.1016/j.ebiom.2020.103077 33166788 PMC7653063

[25] Migueles SA Sabbaghian MS Shupert WL Bettinotti MP Marincola FM Martino L. HLA B*5701 Is Highly Associated With Restriction of Virus Replication in a Subgroup of HIV-Infected Long Term Nonprogressors. Proc Natl Acad Sci USA (2000) 97:2709–14. doi: 10.1073/pnas.050567397 PMC15994 10694578

[26] Pernas M Tarancón-Diez L Rodríguez-Gallego E Gómez J Prado JG Casado C. Factors Leading to the Loss of Natural Elite Control of HIV-1 Infection. J Virol (2018) 92:e01805-17. doi: 10.1128/JVI.01805-17 29212942 PMC5809746

[27] Walker-Sperling VE Pohlmeyer CW Veenhuis RT May M Luna KA Kirkpatrick AR. Factors Associated With the Control of Viral Replication and Virologic Breakthrough in a Recently Infected HIV-1 Controller. EBioMedicine (2017) 16:141–9. doi: 10.1016/j.ebiom.2017.01.034 PMC5474502 28159573

[28] Noel N Lerolle N Lécuroux C Goujard C Venet A Saez-Cirion A. Immunologic and Virologic Progression in HIV Controllers: The Role of Viral “Blips” and Immune Activation in the ANRS CO21 CODEX Study. PloS One (2015) 10:e0131922. doi: 10.1371/journal.pone.0131922 26146823 PMC4493076

[29] Rodríguez-Gallego E Tarancón-Diez L García F Del Romero J Benito JM Alba V. Proteomic Profile Associated With Loss of Spontaneous Human Immunodeficiency Virus Type 1 Elite Control. J Infect Dis (2019) 219:867–76. doi: 10.1093/infdis/jiy599 30312441

[30] Fuertes-Martín R Correig X Vallvé J-C Amigó N. Title: Human Serum/Plasma Glycoprotein Analysis by 1H-NMR, an Emerging Method of Inflammatory Assessment. J Clin Med (2020) 9:354. doi: 10.3390/jcm9020354 PMC7073769 32012794

[31] Kelesidis T Zhang Y Tran E Sosa G Middlekauff HR. Expression of Key Inflammatory Proteins Is Increased in Immune Cells From Tobacco Cigarette Smokers But Not Electronic Cigarette Vapers: Implications for Atherosclerosis. J Am Heart Assoc (2021) 10:e019324. doi: 10.1161/JAHA.120.019324 33356378 PMC7955503

