# Supplementary material for: Evolution of Serum Acute-Phase Glycoproteins Assessed by 1H-NMR in HIV Elite Controllers
Source: Front Immunol. 2021 Sep 28;12:730691. doi: 10.3389/fimmu.2021.730691 (PMC8505996; doi:10.3389/fimmu.2021.730691)
Supplement: Supplementary file 1 [file Table_1.pdf]

Supplementary Material

Article

EVOLUTION OF SERUM ACUTE-PHASE GLYCOPROTEINS ASSESSED BY 1H-NMR IN HIV ELITE CONTROLLERS

Ana-Irene Malo[†], Joaquim Peraire[†], Ezequiel Ruiz-Mateos, Jenifer Masip, Núria Amigó, José Alcamí, Santiago Moreno, Josefa Girona, Graciano García-Pardo, Rosaura Reig, Francesc Vidal, Antoni Castro, Lluís Masana[*‡] and Anna Rull[*‡]

Supplemental Table 1. Plasma glycoproteome analysis in the study group at time point T0.

	PLW-EC		P-value	PLW-TP		Non-HIV	P-value
	**PC (n = 11)**	**TC (n = 11)**	*****	**>350 (n = 11)**	**<100 (n = 11)**	**(n = 11)**	******
Glyc B (µmol/L)	400.8 [356.0-443.5]	404.0 [373.6-462.5]	0.824	446.0 [406.3-487.5]	563.5 [463.2-621.4]	358.8 [320.6-380.2]	**<0.001**
Glyc A (µmol/L)	746.2 [638.7-887.8]	789.1 [718.9-914.2]	0.710	961.9 [827.5-1051.0]	1032 [957.6-1238.0]	691.4 [664.8-755.5]	**<0.001**
H/W Glyc B	5.0 [4.5-5.6]	5.1 [4.7-5.8]	0.824	5.6 [5.1-6.1]	7.1 [5.9-7.8]	4.5 [4.0-4.8]	**<0.001**
H/W Glyc A	18.3 [15.9-20.8]	20.1 [17.7-20.8]	0.331	20.4 [17.1-22.4]	26.1 [21.7-29.8]	15.7 [15.1-16.8]	**<0.001**

T0 in PLWH-ECs is defined by the loss of control in transient controllers (TC) and the initiation of ART in PLWH-TP. Data are presented as median [interquartile range], and compared using non-parametric Mann-Whitney test between PCs and TCs *, and between all PLWH-EC, PLWH-TP and non-HIV subjects with nonparametric Kruskal-Wallis (KW) adjusted by Bonferroni post-hoc approach **. P value < 0.05 was considered significant and is highlighted in bold.

EC, Elite controller; Glyc, glycoprotein; H/W, height/width glycoprotein ratio; PC, Persistent controller; PLWH, Person living with HIV; TC, Transient controller; TP: typical progressor.
